# Vaccine Therapy

**Published:** 1917-02

**Authors:** B. F. Stout


### CLINICAL PATHOLOGY

DR. B. F. STOUT, Editor, 203 Hicks Bldg., San Antonio, Texas

Communications solicited and should be sent direct to Dr. Stout. Copy should be in Dr. Stout's possession by the 20th of the preceding month.

### Vaccine Therapy.

BY B. F. STOUT, M. D.

Everyone interested in modern medicine realizes the great interest manifested in the last few years in vaccines. Thoughtful physicians do not have the faith in drug therapy that they had a decade or so ago, and since vaccine therapy represents certainly a logical attempt to cure disease by applying the principles of immunity it has appealed more to their reason, and a widespread use of these biologic agents has resulted. Many factors entering into the situation make it difficult to measure just how useful they are. Of course, as in the use of any remedy, extreme views are advanced, some being overenthusiastic and others being wholly pessimistic. The middle ground is here the safest, as it so often is in other problems.

Comparatively few who use vaccines have the necessary knowledge of etiology, pathology and immunology to render their observations of much value. Coincidence has caused many a remedy to be credited with undue success. Ignorance of the nature of the remedy has caused unjust criticism.

In view of the confusion existing regarding the value of vaccines, a short review of some recent publications will be of interest. These articles are accessible to anybody, and should be read in the original.

Davis, of Chicago, an investigator of considerable ability and prominence, has written in the *Journal A. M. A.*, No. 3, Vol. LXVII, concerning vaccines from the standpoint of the immunologist. Davis is somewhat pessimistic regarding the curative value of vaccines. He takes up the well known facts connected with the history of therapeutic inoculation and reasons that because so few diseases can be prevented by this method that little more could be expected regarding their cure.

In the instances where vaccines seem to have been of definite benefit, he regards the result as probably due to non-specific reactions. Nearly all observers have seen instances where a severe reaction was followed by distinct benefit by a dose of vaccine, and have attributed the effect to its specific character. Evidence is accumulating to the effect that the results are frequently obtained by non-specific factors.

Typhoid fever, arthritis, including the gonorrheal type, have be cured by crisis, following the intravenous injection of foreign proteins, such as albumose, colon vaccine, horse serum, etc. These injections produce high fever and high leucocytosis with a chill. The explanation of the action of these agents is best explained by quoting from the article under consideration.

Jobling and Peterson, in their analysis of the action of non-specific therapy, call attention to a number of factors. Selective stimulation of the hematopoietic tissues occurs, which results in flooding the body with antibodies which are specific, but the stimulus non-specific. Hyperpyrexia and leucocytosis, and the mobilization of the serum protease and lipase with an ultimate rise of the antiferment titer of the serum following injection of foreign substances are considered significant processes.

Davis believes that where cure and prevention of recurrence is to be aimed at, a specific vaccine is more likely to be of avail. In his summary he states that the non-specific effect of vaccines is now the most important problem that concerns the vaccinationist.

In the same number of the *Journal of the A. M. A.* in which the above was published appears one by Coates, of Philadelphia. He approaches the subject purely from the clinical standpoint, and quotes from his experience in the domain of nose, throat and aural diseases. In acute colds he has had successes in curing them and in preventing the frequent relapses that so many patients have.

In infections of the accessory sinuses after drainage has been satisfactorily provided he believes vaccines to be of value.

He quotes various authors regarding the treatment of ozena, and seems to feel that the evidence is encouraging. Evidence also is presented that vaccines are of distinct value in bronchial asthma. He devotes much space to the suppurative ear troubles, and quotes a series of fifty cases of his own in which vaccines were used and in which dry ears were obtained in 46 per cent. He comments that since all of these would have gone on to radical operation without vaccines, surely the method of treatment has counted for much here. In his summary Coates feels that, while vaccines have not fulfilled all that might be expected of them, they have provided an extra very effective means of combating infection, especially if used in connection with other rational means of treatment.

